# Occupational psychosocial risks as predictors of depression, anxiety, and stress among hospital employees

**DOI:** 10.1371/journal.pone.0340104

**Published:** 2026-01-28

**Authors:** Aaron Siong Fatt Tsen, Khamisah Awang Lukman, Mohammad Saffree Jeffree, Syed Shajee Husain, Izzul Syazwan Ismail

**Affiliations:** 1 Department of Public Health Medicine, Faculty of Medicine and Health Sciences, Universiti Malaysia Sabah, Jalan UMS, Kota Kinabalu, Sabah, Malaysia; 2 Centre for Occupational Safety and Health, Universiti Malaysia Sabah, Jalan UMS, Kota Kinabalu, Sabah, Malaysia,; 3 Faculty of Health, Medicine and Social Care, School of Allied Health and Social Care, Anglia Ruskin University, United Kingdom; 4 Medical Division, Sabah State Health Department, Rumah Persekutuan, Jalan Mat Salleh, Kota Kinabalu, Sabah, Malaysia; Universiti Pertahanan Nasional Malaysia, MALAYSIA

## Abstract

Workplace mental health is a growing concern in Malaysia’s healthcare sector, yet comprehensive psychosocial risk assessments across all staff remain limited. This cross-sectional study examined the prevalence and predictors of depression, anxiety, and stress among employees in four government tertiary hospitals in Kota Kinabalu, namely Hospital Queen Elizabeth, Hospital Queen Elizabeth II, Hospital Wanita dan Kanak-Kanak Sabah, and Hospital Mesra Bukit Padang. From 21^st^ March 2025–20^th^ April 2025, 233 staff members were selected via stratified random sampling. Data were collected using validated self-administered online questionnaires, including the 21-item Depression, Anxiety and Stress Scale and the Likelihood of Environment & Occupational Exposure Scale towards Psychosocial Risk in the Workplace. Analyses involved descriptive statistics, bivariate comparisons, and multivariate logistic regression using SPSS version 29. Results revealed high prevalence rates of anxiety (43.8%), depression (37.8%), and stress (27.0%). Bivariate analysis revealed elevated odds of depression among Chinese ethnicity, diploma educated, high-income staff, HQE employees, medical and clinical roles, doctors, and shift workers. Anxiety was linked to medical departments and shift work, while stress was prevalent in younger staff with shorter tenure. High job demand, low control, and inadequate support increased depression, anxiety, and stress risk. Multivariate analysis identified high psychosocial risks related to job demand (OR 3.94), control (OR 3.72), and support (OR 2.87) as significant predictors of depression. High psychosocial risk in job demand (OR 3.01), control (OR 2.29), and support (OR 2.59) also predicted anxiety. Stress was closely linked to staff aged 20–39 years (OR 3.14), high psychosocial risk in job control (OR 4.45), and support (OR 2.68). Although the cross-sectional design and reliance on self-report limit causal interpretation, these findings highlight the value of regular psychosocial risk assessments and targeted interventions. Strengthening workplace support systems is crucial to improving mental well-being among Malaysia’s hospital workforce.

## Introduction

In the post-pandemic era, following the COVID-19 crisis, workplace mental health has become an urgent and enduring public health concern, drawing the attention of both global and national stakeholders. Across the world, depression and anxiety are among the leading contributors to lost productivity, with estimates suggesting that up to 15% of the working-age population suffer from mental health conditions that account for billions in lost working days and economic output each year [1–2]. In Malaysia, these trends are mirrored acutely within the healthcare sector, where recent studies have found that rates of depression, anxiety, and stress among clinical and non-clinical staff can reach as high as 18.7%, 38.6%, and 12%, respectively [3–5].

Certain groups within hospital settings are especially at risk: for instance, female healthcare providers have about twice the odds of experiencing depression and anxiety compared to their male counterparts [6], while younger staff and those from lower-income backgrounds report markedly elevated rates of psychological symptoms [7]. Notably, shift work and irregular schedules have also been shown to intensify conditions such as anxiety and sleep disturbances among hospital workers [8]. Psychosocial hazards, in particular high job demands and low job control, consistently emerge as key predictors of distress, while inadequate workplace support further amplifies vulnerability [9]. The theoretical underpinning for this research lies in the Job Demand-Control-Support models, which explain how exposure to high demands, low job control, and insufficient social support elevate the risk of depression, anxiety, and stress [10–12].

Recognizing these ongoing challenges, the Malaysian government has introduced substantial reforms, including amendments to the Occupational Safety and Health Act and updated psychosocial risk assessment protocols [13]. However, scientific inquiry in this field has often been narrow in focus, with much of the research centered primarily on doctors and nurses, neglecting many other key staff groups and rarely employing standardized, comprehensive tools for psychosocial assessment. This limited perspective constrains the understanding of the full burden and landscape of mental health risks, particularly in tertiary hospitals and less-studied areas such as Sabah [14–15].

To address these critical gaps, the present study provides a comprehensive assessment of mental health among all categories of government tertiary hospital employees in Kota Kinabalu. Specifically, this research seeks to determine the prevalence of depression, anxiety, and stress, and to identify the associated sociodemographic, occupational, and psychosocial risk due to work factors. By expanding the scope of inquiry and utilizing validated, robust measurement tools, the study will produce essential evidence for the development of effective, targeted interventions and equitable policies supporting the mental well-being of Malaysia’s healthcare workforce.

## Materials and methods

### Study design and setting

This cross-sectional study was conducted from 21st March 2025–20th April 2025 among employees of four government tertiary hospitals in Kota Kinabalu, Sabah: Hospital Queen Elizabeth (HQE), Hospital Queen Elizabeth II (HQE II), Hospital Wanita dan Kanak-Kanak Sabah (HWKKS), and Hospital Mesra Bukit Padang (HMBP). The selection of these four hospitals was purposive, as they represent the complete set of government tertiary care facilities within the Kota Kinabalu region.

### Participants and recruitment

Collectively, the staff establishment of these hospitals comprises 44% of the total 18,674 personnel in the Medical Division of the Sabah State Health Department. Eligible participants were all staff who had worked in the selected hospitals for at least six months. Individuals on leave or with a diagnosed mental health condition were excluded. Stratified random sampling was employed to ensure proportional representation across hospitals and staff categories, namely management and professional, paramedic and auxiliary, and supporting groups. The minimum sample size was 212, calculated based on difference-in-proportion estimates calculated using G*Power software, targeting a 90% power and 0.05 error rate [16–17]. Adjusted sample size was 265 for an anticipated 20% non-response rate [7]. A comprehensive sampling frame was established using up-to-date staff lists from each hospital.

### Data collection

Data were collected through a self-administered, web-based questionnaire (Google Forms). The instrument comprised three main sections: (1) sociodemographic data, including age, sex, ethnicity, marital status, education, and household income; (2) occupational characteristics, such as hospital, department, job position, work type, experience, contract status, and shift duty, along with the Likelihood of Environment & Occupational Exposure Scale towards Psychosocial Risk in the Workplace (LEO26); and (3) the validated 21-item Depression, Anxiety, and Stress Scale (DASS-21). Psychosocial risk was assessed using the LEO26, which evaluates job demand, control, and support on a 5-point Likert scale. Based on established criteria, the thresholds for high psychosocial risk were delineated as scores of 7.5 or higher for job demand, 15.5 or higher for job control, and 38.5 or lower for job support, with scores falling outside these respective ranges indicating low risk [18–19]. Mental health outcomes were measured with the DASS-21. Following protocol, participants were classified for the presence (“Yes”) of symptoms if their adjusted scores exceeded 9 for depression, 7 for anxiety, and 14 for stress, whereas scores at or below these respective cut-offs indicated an absence (“No”) of clinically significant symptoms [20].

### Data analysis and management

Completed questionnaires were exported to IBM Statistical Product and Service Solution (SPSS) software version 29 for analysis. Descriptive statistics were reported as frequencies, percentages, means, or medians as appropriate. Associations between independent variables (sociodemographic, occupational, and psychosocial risk due to work factors) and dependent variables (depression, anxiety, and stress) were examined using simple logistic regression. Factors meeting p < 0.05 or considered clinically relevant at p < 0.25 were included in multiple logistic regression models. Odds ratios (OR), 95% confidence intervals (CI), and p-values were reported.

Data integrity was ensured by managing missing data with listwise deletion or mean imputation where necessary. The evaluation of model fit involved checking for the absence of multicollinearity and interaction effects to ensure model stability. The Hosmer–Lemeshow test was conducted to assess the goodness of fit, requiring a non-significant result (p > 0.05) to indicate adequate model calibration. Classification accuracy was determined using a cut-off point of 0.5 for predicted probabilities, while the discriminatory ability of the model was evaluated by calculating the area under the receiver operating characteristic (ROC) curve with a threshold of 0.7 to indicate acceptable performance.

### Ethical consideration

This study was conducted in full accordance with the Declaration of Helsinki. Ethical approval for the research protocol, including the informed consent procedures, was obtained from the Medical Research and Ethics Committee of the Malaysian Ministry of Health [Ref: NMRR ID-24–04090-LTE, 10^th^ February 2025] and the Universiti Malaysia Sabah Research Ethics Committee [Ref: JKEtika 2/25(11), 20^th^ March 2025].

Following ethics approval, participant recruitment occurred from 21^st^ March 2025–20^th^ April 2025. Prior to any data collection, all individuals were provided with a participant information sheet detailing the study’s objectives. Subsequently, each participant provided written informed consent by signing the ethics-approved consent form. Confidentiality and anonymity were strictly maintained throughout the study; all data were securely stored by, and accessible only to, the principal investigator.

## Results

### Descriptive analysis

A total of 233 employees (response rate: 87.9%) from four government tertiary hospitals in Kota Kinabalu participated in this study, ensuring adequate statistical power. The sample was predominantly female (73.8%) and young (mean age 38.8 years, 60.9% aged 20–39), with the vast majority identifying as Bumiputera Sabah (75.1%) and married (75.5%). Most respondents held at least a diploma or STPM (43.8%), and nearly half reported a middle household income (Table 1).

**Table 1:** Sociodemographic factors and prevalence of depression, anxiety, stress (n = 233).

Variables	n	(%)
**SOCIODEMOGRAPHIC FACTORS**		
**Age (years)**		
20–39 years old	142	(60.9)
40–59 years old	91	(39.1)
**Sex**		
Male	61	(26.2)
Female	172	(73.8)
**Ethnicity**		
Bumiputera Sabah	175	(75.1)
Malay	31	(13.3)
Chinese	14	(6.0)
Others	13	(5.6)
**Marital Status**		
Married	176	(75.5)
Never Married	57	(24.5)
**Education level**		
PMR and SPM	51	(21.9)
Diploma and STPM	102	(43.8)
Degree and above	80	(34.3)
**Household Income (RM)**		
Low (<RM 3,840)	48	(20.6)
Middle (RM 3,840 – RM 8,529)	107	(45.9)
High (≥ RM 8,530)	78	(33.5)
**PREVALENCE OF DEPRESSION, ANXIETY, STRESS**		
**Depression**		
No	145	(62.2)
Yes	88	(37.8)
**Anxiety**		
No	131	(56.2)
Yes	102	(43.8)
**Stress**		
No	170	(73.0)
Yes	63	(27.0)

Over half worked at Hospital Queen Elizabeth, and about four-fifths were in clinical roles; shift or on-call work was reported by 71.2% of participants. Regarding psychosocial risk, 86.7% had high job demand, 76.4% high risk due to low job control, and about half reported low job support. Mental health symptoms were highly prevalent: 43.8% met criteria for anxiety, 37.8% for depression, and 27.0% for stress. (Table 2).

**Table 2:** Occupational characteristics and psychosocial risk due to work factors (n = 233).

Variables	n	(%)
**OCCUPATIONAL CHARACTERISTICS**		
**Hospital**		
Hospital Queen Elizabeth	128	(54.9)
Hospital Queen Elizabeth II	37	(15.9)
Hospital Wanita dan Kanak-Kanak Sabah	49	(21.0)
Hospital Mesra Bukit Padang	19	(8.2)
**Work Department**		
Medical-Based	115	(49.4)
Surgical-Based	38	(16.3)
Clinical Support	30	(12.9)
Non-Clinical Support	50	(21.5)
**Service Group**		
Management and Professionals	59	(25.3)
Paramedics and Auxiliary	140	(60.1)
Support Group	34	(14.6)
**Job Position**		
Doctor	42	(18.0)
Nurse	96	(41.2)
Other Paramedic/ Auxiliary Staffs	57	(24.5)
Admin/ Other Non-Clinical Staffs	39	(16.3)
**Work Type**		
Non-clinical	48	(20.6)
Clinical	185	(79.4)
**Grade**		
Low (1–4)	59	(25.3)
Middle (5–8)	119	(51.1)
High (9 and above)	55	(23.6)
**Work Experience (Years)**		
5 years and below	31	(13.3)
6–10 years	44	(18.9)
11–15 years	92	(39.5)
16 years and above	66	(28.3)
**Employment Contract Status**		
Permanent	224	(96.1)
Contract	9	(3.9)
**Shift Work/ Oncall**		
No	67	(28.8)
Yes	166	(71.2)
**PSYCHOSOCIAL RISK DUE TO WORK FACTORS**		
**Psychosocial Risk due to Job Demand**		
Low Risk	31	(13.3)
High Risk	202	(86.7)
**Psychosocial Risk due to Job Control**		
Low Risk	55	(23.6)
High Risk	178	(76.4)
**Psychosocial Risk due to Job Support**		
Low Risk	115	(49.4)
High Risk	118	(50.6)

### Bivariate analysis

Bivariate analyses revealed that Chinese ethnicity (OR=3.28, 95% CI: 1.05–10.22), diploma/STPM education (OR=2.47, 95% CI: 1.16–5.25), and high income (OR=2.19, 95% CI: 1.01–4.77) group were each associated with higher odds of depression. Stress was more likely in younger staff (OR=3.26, 95% CI: 1.65–6.44) (Table 3).

**Table 3:** Simple logistic regression: Sociodemographic factors and depression, anxiety, stress.

Variables	Depression	Anxiety	Stress
	OR (95% CI)	OR (95% CI)	OR (95% CI)
**Age**			
40–59 years old	1.00	1.00	1.00
20–39 years old	1.40 (0.81, 2.43)	1.66 (0.97, 2.85)	**3.26 (1.65, 6.44)**
**Sex**			
Male	1.00	1.00	1.00
Female	1.10 (0.60, 2.02)	1.40 (0.77, 2.55)	1.51 (0.76, 3.03)
**Ethnicity**			
Bumiputera Sabah	1.00	1.00	1.00
Malay	1.15 (0.52, 2.53)	1.10 (0.51, 2.37)	1.42 (0.62, 3.24)
Chinese	**3.28 (1.05, 10.22)**	1.33 (0.45, 3.96)	1.65 (0.53, 5.20)
Others	1.14 (0.36, 3.63)	1.14 (0.37, 3.54)	1.32 (0.39, 4.51)
**Marital Status**			
Married	1.00	1.00	1.00
Never Married	1.54 (0.84, 2.83)	0.91 (0.50, 1.67)	1.67 (0.88, 3.18)
**Education level**			
PMR and SPM	1.00	1.00	1.00
Diploma and STPM	**2.47 (1.16, 5.25)**	1.37 (0.70, 2.71)	1.63 (0.72, 3.68)
Degree and above	2.17 (0.99, 4.76)	0.90 (0.44, 1.85)	1.76 (0.76, 4.07)
**Household Income**			
Low (<RM 3,840)	1.00	1.00	1.00
Middle (RM 3,840 – RM 8,529)	1.61 (0.76, 3.39)	1.02 (0.51, 2.03)	1.61 (0.70, 3.74)
High (≥ RM 8,530)	**2.19 (1.01, 4.77)**	1.26 (0.61, 2.61)	2.04 (0.86, 4.86)

Depression was consistently reported among staff at Hospital Queen Elizabeth (OR=6.20, 95% CI: 1.38–28.00), those in medical-based (OR=2.93, 95% CI: 1.36–6.28), clinical roles (OR=2.77, 95% CI: 1.30–5.90), doctors (OR=3.54, 95% CI: 1.35–9.28), and shift/on-call workers (OR=2.12, 95% CI: 1.17–4.13). Anxiety was linked with medical-based work department (OR=2.19, 95% CI: 1.10–4.37) and shift works (OR=1.90, 95% CI: 1.05–3.45). For stress, having six to ten years (OR=5.28, 95% CI: 2.10–13.24) or fewer (OR=3.02, 95% CI: 1.08–8.45) work experience notably raised risk (Table 4).

**Table 4:** Simple logistic regression: Occupational characteristic and depression, anxiety, stress.

Variables	Depression	Anxiety	Stress
	OR (95% CI)	OR (95% CI)	OR (95% CI)
**Hospital**			
Hospital Mesra Bukit Padang	1.00	1.00	1.00
Hospital Queen Elizabeth	**6.20 (1.38, 28.00)**	2.63 (0.90, 7.73)	1.30 (0.40, 4.21)
Hospital Queen Elizabeth II	4.08 (0.81, 20.59)	1.52 (0.45, 5.16)	1.03 (0.67, 4.00)
Hospital Wanita dan Kanak-Kanak Sabah	**5.86 (1.22, 28.23)**	2.28 (0.71, 7.32)	2.18 (0.63, 7.57)
**Work Department**			
Non-Clinical Support	1.00	1.00	1.00
Medical-Based	**2.93 (1.36, 6.28)**	**2.19 (1.10, 4.37)**	1.89 (0.87, 4.09)
Surgical-Based	2.31 (0.91, 5.88)	1.27 (0.53, 3.04)	0.81 (028, 2.31)
Clinical Support	1.77 (0.64, 4.88)	0.83 (0.31, 2.21)	0.71 (0.22, 2.29)
**Service Group**			
Support Group	1.00	1.00	1.00
Paramedics and Auxiliary	2.04 (0.86, 4.83)	1.91 (0.87, 4.23)	1.80 (0.69, 4.69)
Management and Professionals	2.56 (0.99, 6.59)	1.43 (0.59, 3.48)	2.05 (0.72, 5.81)
**Job Position**			
Admin/ Other Non-Clinical Staffs	1.00	1.00	1.00
Doctor	**3.54 (1.35, 9.28)**	1.97 (0.79, 4.91)	2.46 (0.87, 6.93)
Nurse	2.21 (0.94, 5.17)	1.83 (0.83, 4.05)	1.73 (0.68, 4.41)
Other Paramedic/ Auxiliary Staffs	1.49 (0.59, 3.78)	1.82 (0.77, 4.29)	1.44 (0.52, 3.99)
**Work Type**			
Non-clinical	1.00	1.00	1.00
Clinical	**2.77 (1.30, 5.90)**	1.95 (0.99, 3.84)	1.79 (0.81, 3.94)
**Grade**			
Low (1–4)	1.00	1.00	1.00
Middle (5–8)	**2.25 (1.12, 4.54)**	1.25 (0.67, 2.36)	1.75 (0.81, 3.76)
High (9 and above)	**2.68 (1.20, 5.97)**	1.05 (0.50, 2.21)	2.12 (0.90, 5.04)
**Work Experience**			
16 years and above	1.00	1.00	1.00
11–15 years	1.17 (0.60, 2.28)	1.04 (054, 1.98)	2.24 (0.96, 5.19)
6–10 years	1.52 (0.69, 3.34)	1.85 (0.85, 4.00)	**5.28 (2.10, 13.24)**
5 years and below	1.44 (0.60, 3.48)	1.44 (0.61, 3.41)	**3.02 (1.08, 8.45)**
**Employment Contract Status**			
Permanent	1.00	1.00	1.00
Contract	2.12 (0.56, 8.13)	1.03 (0.27, 3.93)	2.24 (0.58, 8.61)
**Shift Work/ On-call**			
No	1.00	1.00	1.00
Yes	**2.12 (1.17, 4.13)**	**1.90 (1.05, 3.45)**	1.41 (0.73, 2.74)

Elevated psychosocial risks across key work-related factors were significantly associated with higher odds of depression, anxiety, and stress. Specifically, high job demand was linked to markedly increased odds of depression (OR = 6.78; 95% CI: 2.00–23.04), anxiety (OR = 4.84; 95% CI: 1.77–13.00), and stress (OR = 6.27; 95% CI: 1.45–27.12) compared to low job demand. Similarly, high risk due to low job control emerged as a potent predictor, with odds of depression (OR = 6.98; 95% CI: 2.84–17.12), anxiety (OR = 4.18; 95% CI: 2.03–8.62), and stress (OR = 6.32; 95% CI: 2.18–18.33) substantially higher than in the low-risk group. Moreover, insufficient job support significantly elevated the likelihood of depression (OR = 4.12; 95% CI: 2.33–7.31), anxiety (OR = 3.45; 95% CI: 2.03–6.04), and stress (OR = 4.10; 95% CI: 2.15–7.80) (Table 5).

**Table 5:** Simple logistic regression: Psychosocial risk due to work factors and depression, anxiety, stress.

Variables	Depression	Anxiety	Stress
	OR (95% CI)	OR (95% CI)	OR (95% CI)
**Psychosocial Risk due to Job Demand**			
Low Risk	1.00	1.00	1.00
High Risk	**6.78 (2.00, 23.04)**	**4.84 (1.77, 13.00)**	**6.27 (1.45, 27.12)**
**Psychosocial Risk due to Job Control**			
Low Risk	1.00	1.00	1.00
High Risk	**6.98 (2.84, 17.12)**	**4.18 (2.03, 8.62)**	**6.32 (2.18, 18.33)**
**Psychosocial Risk due to Job Support**			
Low Risk	1.00	1.00	1.00
High Risk	**4.12 (2.33, 7.31)**	**3.45 (2.03, 6.04)**	**4.10 (2.15, 7.80)**

### Multivariate analysis

Multivariate models, where Forward and Backward Logistic Regression (LR) methods were applied, confirmed psychosocial work factors as the most robust predictors. After adjustment, high psychosocial risk due to job demand (aOR=3.94, 95% CI: 1.08–14.39), job control (aOR=3.72, 95% CI: 1.44–9.63), and job support (aOR=2.87, 95% CI: 1.56–5.26) remained significantly associated with depression. For anxiety, the same factors remained influential, with adjusted odds ratios of 3.01 (95% CI: 1.04–8.66) for high psychosocial risk due to job demand, 2.29 (95% CI: 1.04–5.06) for job control, and 2.59 (95% CI: 1.45–4.63) for poor support. Stress was predicted independently by younger age (aOR=3.14, 95% CI: 1.54–6.40), high psychosocial risk due to job control (aOR=4.45, 95% CI: 1.45–13.66), and job support (aOR=2.68, 95% CI: 1.35–5.34) (Table 6).

**Table 6:** Multiple logistic regression: Factors associated with depression, anxiety, stress.

Variables	Depression	Anxiety	Stress
	aOR (95% CI)	aOR (95% CI)	aOR (95% CI)
**Psychosocial Risk due to Job Demand**			
Low Risk	1.00	1.00	--
High Risk	**3.94 (1.08, 14.39)**	**3.01 (1.04, 8.66)**	
**Psychosocial Risk due to Job Control**			
Low Risk	1.00	1.00	1.00
High Risk	**3.72 (1.44, 9.63)**	**2.29 (1.04, 5.06)**	**4.45 (1.45, 13.66)**
**Psychosocial Risk due to Job Support**			
Low Risk	1.00	1.00	1.00
High Risk	**2.87 (1.56 5.26)**	**2.59 (1.45, 4.63)**	**2.68 (1.35, 5.34)**
**Age**			
40–59 years old	--	--	1.00
20–39 years old			**3.14 (1.54, 6.40)**

**Fig 1:**
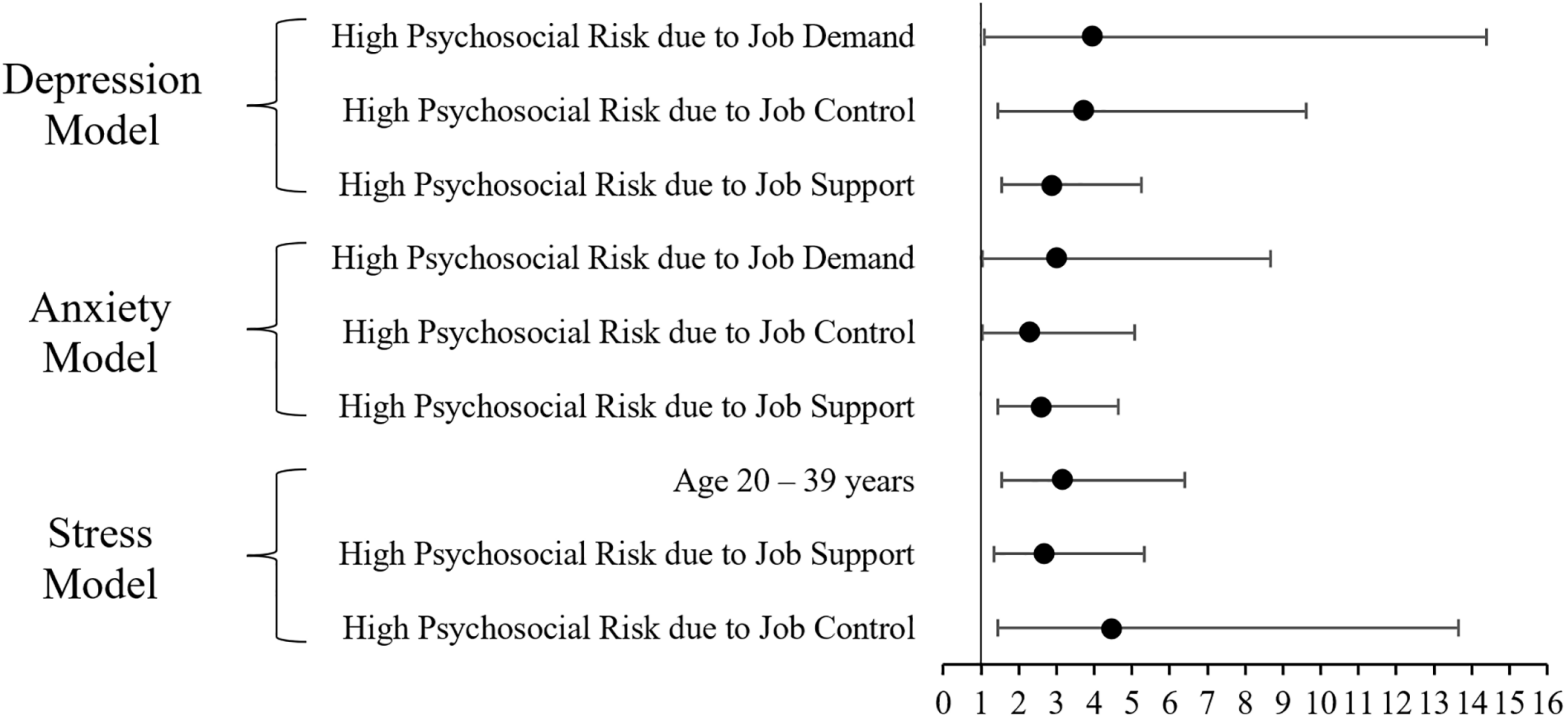
Adjusted Odds Ratios for Predictors of Depression, Anxiety, and Stress.

Based on the Forest Plot (Fig 1), the multivariate analysis clearly establishes that psychosocial work factors are the most dominant and reliable predictors of all three mental health outcomes in the hospital workforce. Specifically, high psychosocial risk due to low job control emerges as the strongest risk factor, showing the highest aOR for both stress and depression. High job demand and inadequate job support also significantly increase the odds across depression and anxiety models, with all corresponding 95% CI not crossing the line of no effect. In contrast to the psychosocial factors, only age (20–39 years) retains significance in the final multivariate model, acting as an independent predictor solely for stress.

Assessment for multicollinearity and interaction effects showed no issues, ensuring model stability. Model diagnostics indicated good fit, with the Hosmer–Lemeshow test results (Depression: p = 0.997; Anxiety: p = 0.800; Stress: p = 0.446) showing no significant differences between observed and predicted values. The overall classification accuracy for the predictive models was 69.1% for depression (compared to 62.2% for the constant model), 67.4% for anxiety (compared to 56.2% for the constant model), and 73.4% for stress (compared to 73.0% for the constant model). The discriminatory ability of the models, as assessed by the Area Under the Receiver Operating Characteristic (ROC) curve, confirmed acceptable predictive performance and fair discrimination: Depression 0.73 (95% CI: 0.65–0.79), Anxiety 0.70 (95% CI: 0.63–0.77), and Stress 0.74 (95% CI: 0.68, 0.81) (Table 7).

**Table 7:** Model fit assessment.

Assessment	Depression	Anxiety	Stress
**Hosmer and Lemeshow Test**	p = 0.997	p = 0.8	p = 0.446
**Classification table**	Constant Model: 62.2% Predictive Model: 69.1%	Constant Model: 56.2% Predictive Model: 67.4%	Constant Model: 73.0% Predictive Model: 73.4%
**Area Under ROC Curve**	0.73 (95% CI: 0.65–0.79)	0.70 (95% CI: 0.63–0.77)	0.74 (95% CI: 0.68, 0.81)

## Discussion

In Kota Kinabalu tertiary hospitals, the healthcare workforce is mainly young, predominantly female in nursing and allied health but male among doctors and medical assistants, and mainly Bumiputera Sabah, reflecting both recent recruitment drives and the demography of the population. Most are married (69.4%) and possess role-appropriate educational qualifications (diplomas for nurses or other allied health professionals, degrees for doctors or pharmacists) and fall within the government-defined middle-income bracket, with variations between occupational groups [21–23]. The clinical focus, especially in major hospitals, results in a staff structure weighted towards clinical roles, permanent employment, and shift work, but with a hierarchical system that limits advancement [24–25]. The psychosocial risk landscape is marked by elevated job demand, well above previous Malaysian reports, and driven by persistent staff shortages, intense workloads, and extensive shift requirements, which are especially relevant for younger, largely female staff [26–28]. Job control is generally low due to protocol-driven settings, and support systems are inconsistently robust, with more than half of staff at high psychosocial risk for low support [29–30].

In this study, the bivariate analysis showed Chinese ethnicity staff had a higher odd for depression (OR=3.28, 95% CI: 1.05–10.22) compared to Bumiputera Sabah. However, the interpretation of this specific finding must be viewed as exploratory. Given that the Chinese subgroup represented a small sample size (n = 14) and the confidence interval is very wide, this estimate is inherently uncertain. The unequal and small group sizes result in reduced statistical power, which limits the generalizability of this particular association.

Globally, substantial international evidence underpins that high job demands dramatically raise depression odds (OR = 4.34 in Korea, OR = 3.33 in the Philippines), with risk escalating as work hours increase [31–33]. The stress-response model explains that chronic high job demand produces sustained psychological strain and neuroendocrine changes that precipitate depressive symptoms [34]. Conversely, some population-based studies, such as those from Sweden, report weaker or even reversed associations when job demand is evaluated alongside high control and support, an effect captured by the demand-control-support model, which posits that high demand may foster satisfaction if autonomy and support are sufficient [35]. Job control and autonomy consistently offer robust protection; limited decision-making increases depression (HR up to 1.43 in men, 1.27 in women), especially with chronic exposure [36–37]. These effects are mediated by self-efficacy, motivation, and perceptions of helplessness [38]. In parallel, low job support markedly raises depression risk, while strong peer and supervisor relationships, as well as a positive psychosocial safety climate, reduce risk [39–41]. Still, these supports are most effective when combined with personal resilience, as shown in European analyses [42].

In this study population, anxiety prevalence was closely resembled rates from those reported in global meta-analyses, but significantly exceeding rates reported for the Northwest region of Malaysia [43–45]. The elevated anxiety reflects the intense psychological challenge of hospital environments, characterized by high workloads, patient acuity, irregular shifts, and lack of support [46–47]. Cognitive overload, the theory that chronic excessive demand depletes coping resources and impairs decision-making, is supported by findings linking sustained workload, understaffing, and unpredictable hours to anxiety, as well as emotional exhaustion and anticipatory worry [48–49]. This relationship is further shaped by interconnected psychosocial factors, including role ambiguity, unsafe working conditions, career development pressures, and contextual moderators such as organizational trust, gender, and individual coping styles, underscoring the need to view job control within a broader, multifactorial framework of workplace mental health [50–52]. Social support, clarity in obligations, and job control all buffer against adverse effects, but their absence exacerbates cognitive overload and prevents recovery [53–54]. Internationally, similar psychosocial conditions produce comparable anxiety patterns in different occupational characteristics: emergency physicians in Turkey report extremely high risk (OR = 5.583), while nurses and other staff in the Philippines and Europe also show strong associations with precarious work, shift assignment, and high job insecurity [55–57]. However, in this analysis, neither job role, shift, nor contract type retained significance once psychosocial exposures were considered, underscoring the primacy of the immediate work environment over background variables.

Stress was particular vulnerability of early-career professionals to adverse work environments. Multiple studies affirm younger staff experience three times higher odds of stress, exacerbated by increased demands, poor recovery, and underdeveloped coping skills [58–59]. Extended working hours, rigid schedules, and shift intensity further increase stress and risk of burnout, while other sociodemographic and job attributes become less predictive after controlling for psychosocial risks [60–62]. The Challenge-Hindrance-Threat appraisal framework contextualizes this, differentiating challenge stressors (which may motivate) from hindrances and threats (role ambiguity, lack of support), with the latter being especially linked to stress [63]. In line with Lazarus and Folkman’s transactional model of stress, the experience and impact of stress result from the appraisal of environmental demands relative to available resources. When demands outweigh coping resources, such as autonomy and support, stress responses are triggered, giving rise to both emotional exhaustion and disengagement [64–65]. Organizational support, peer relationships, and clear role expectations act as critical moderators, buffering stress by 56% via improved coping and appraisal processes [66–67]. However, under conditions of high stigma and psychological distress, the protective value of support is diminished [68].

This study’s exceptional strength lies in its rigorous and contextually sensitive methodology, which integrates a validated psychosocial risk assessment framework compliant with international standards. Through robust methods including stratified random sampling for representativeness, an adequate sample size for statistical power, and sophisticated multivariate logistic regression for precise analysis, the research provides reliable and actionable findings.

The findings of this research, while informative, are subject to several limitations that warrant careful consideration. Its cross-sectional design precludes causal inference regarding psychosocial hazards and mental health outcomes [69]. The use of stratified sampling without applying appropriate weighting to the strata proportions may introduce bias in the estimates, thereby limiting the generalizability of the findings to the broader population. Practical challenges in workforce classification could introduce selection bias and affect subgroup analyses. The absence of adjustment for multiple testing (Bonferroni correction) increases the chance of type I errors in bivariate analyses [70]. Reliance on self-reported data, including the use of the DASS-21 screening tool, risks both underreporting due to social desirability and stigma, as well as misclassification of diagnostic status [71]. Non-response bias may further underestimate prevalence rates, as those in greatest distress may be less likely to participate [72]. Although adjustments were made for measured confounders, residual confounding cannot be fully excluded, particularly from unmeasured coping strategies, institutional culture, or life events [73].

To address these findings, hospitals should routinely implement validated psychosocial risk assessments and translate results into targeted organizational interventions that balance workloads, enhance job control, and strengthen supportive practices throughout all staff levels [74]. Multi-tiered interventions should combine prevention, through workload management and supportive leadership, with early identification of high-risk groups and accessible mental health resources, integrated into core institutional policy [75–76]. At the policy level, requirements for routine psychosocial risk evaluations and accessible reporting mechanisms should be embedded within occupational health regulations, with transparent follow-up and resource allocation to ensure effective implementation [77–78]. For future research, employing advanced methodologies such as Structural Equation Modelling or Generalized Estimating Equations will help clarify the complex interactions between workplace risks and mental health outcomes [79–80]. Ensuring large, diverse samples and consistent use of validated instruments will improve reliability and inform effective, evidence-based interventions [81–82]. Longitudinal and qualitative designs are also recommended to further understand causal relationships and the real-world impact of organizational changes [83–85].

## Conclusion

In conclusion, this study elucidates the substantial burden of depression, anxiety, and stress among personnel in government tertiary hospitals in Kota Kinabalu, with psychosocial work factors identified as predominant determinants. Specifically, heightened job demands, diminished job control, and inadequate workplace support were found to exert a markedly greater influence on mental health outcomes than individual sociodemographic or occupational attributes. These findings underscore the necessity of routine psychosocial risk assessments and the formulation of comprehensive, multi-level interventions that encompass both policy and organizational reforms. Such initiatives should prioritize the enhancement of supportive work environments, the promotion of employee autonomy, and the facilitation of work–life balance. This research provides a critical evidence base to guide targeted strategies and policy reforms aimed at safeguarding and strengthening the mental health and resilience of healthcare workers.
